# The neurophysiological approach to misophonia: Theory and treatment

**DOI:** 10.3389/fnins.2023.895574

**Published:** 2023-03-23

**Authors:** Pawel J. Jastreboff, Margaret M. Jastreboff

**Affiliations:** ^1^Department Otolaryngology, Emory University School of Medicine, Atlanta, GA, United States; ^2^Jastreboff Hearing Disorders Foundation (JHDF), Inc., Ellicott City, MD, United States

**Keywords:** misophonia, hyperacusis, decreased sound tolerance, tinnitus, subconscious conditioned reflexes, limbic system, autonomic nervous system, definitions

## Abstract

Clinical observations of hundreds of patients who exhibited decreased tolerance to sound showed that many of them could not be diagnosed as having hyperacusis when negative reactions to a sound depend only on its physical characteristics. In the majority of these patients, the physical characteristics of bothersome sounds were secondary, and patients were able to tolerate other sounds with levels higher than sounds bothersome for them. The dominant feature determining the presence and strength of negative reactions are specific to a given patient's patterns and meaning of bothersome sounds. Moreover, negative reactions frequently depend on the situation in which the offensive sound is presented or by whom it is produced. Importantly, physiological and emotional reactions to bothersome sounds are very similar (even identical) for both hyperacusis and misophonia, so reactions cannot be used to diagnose and differentiate them. To label this non-reported phenomenon, we coined the term misophonia in 2001. Incorporating clinical observations into the framework of knowledge of brain functions allowed us to propose a neurophysiological model for misophonia. The observation that the physical characterization of misophonic trigger was secondary and frequently irrelevant suggested that the auditory pathways are working in identical manner in people with as in without misophonia. Descriptions of negative reactions indicated that the limbic and sympathetic parts of the autonomic nervous systems are involved but without manifestations of general malfunction of these systems. Patients with misophonia could not control internal emotional reactions (even when fully realizing that these reactions are disproportionate to benign sounds evoking them) suggesting that subconscious, conditioned reflexes linking the auditory system with other systems in the brain are the core mechanisms of misophonia. Consequently, the strength of functional connections between various systems in the brain plays a dominant role in misophonia, and the functional properties of the individual systems may be perfectly within the norms. Based on the postulated model, we proposed a treatment for misophonia, focused on the extinction of conditioned reflexes linking the auditory system with other systems in the brain. Treatment consists of specific counseling and sound therapy. It has been used for over 20 years with a published success rate of 83%.

## 1. Introduction

### 1.1. General comments

The concept of misophonia was first proposed in 2001 (Jastreboff and Jastreboff, 2001a,b), defined as a disorder characterized by abnormally strong negative reactions to patterns of sound specific for a given patient (a full definition of misophonia is presented in Section 4). Importantly, this definition did not assume any specific etiology or mechanism of misophonia.

During the last decade, another approach to misophonia has been proposed, postulating that misophonia is a psychiatric disorder. While the same name, misophonia, was used, it was defined in an entirely different manner. This started confusion as to what misophonia is, which led to an inconsistent selection of study participants, influenced proposed treatments, and had an impact on the criteria used for the outcome measures. Consequently, this significantly hindered the progress of research and development of effective methods of misophonia treatments.

The proposed 2001 definition of misophonia distinguished misophonia from hyperacusis, which causes similar or even identical negative reactions evoked by sound. However, hyperacusis is clearly a separate problem of sound tolerance, and it most likely involves different mechanisms from misophonia and requires specific treatment (Henry et al., 2022). Other existing definitions of misophonia do not consider hyperacusis. Consequently, this negatively affects conclusions and influences future studies of misophonia since people with hyperacusis are not excluded from groups of subjects researched or treated for misophonia. The most recent attempt to reach a consensus regarding the definition of misophonia resulted in an article representing the opinion of a group of experts (Swedo et al., 2022); however, the issue of hyperacusis was disregarded.

This study focuses on clarifying some misconceptions and controversies related to misophonia. The primary goals of the study are as follows: (1) To describe the origin of the concept and supporting data leading to the definition of misophonia as proposed in 2001 and discuss problems arising from incorrectly using the literal translation of the individual components of the new word “misophonia” to define this phenomenon and (2) to summarize the main observations accumulated during more than 30 years of treatment of patients with decreased sound tolerance in clinical practice, which justifies the proposed approach to diagnose misophonia and differentiate it from hyperacusis.

The secondary goals of the study are to present the justification of a potential model and the mechanism of misophonia. The model resulted from combining observations obtained from patients and the basic knowledge of neuroscience. Furthermore, the article briefly outlines treatment based on the neurophysiological model of tinnitus and decreased sound tolerance, known as tinnitus retraining therapy (TRT), modified to include treatment for misophonia as well. The reported high clinical effectiveness of this treatment supports the proposed mechanism of misophonia. At the end of the article, directions and specific projects for future work are outlined.

All presented data and results of the treatment come from the clinical population of patients seeking help for bothersome decreased sound tolerance. All rules applicable to clinical work have been followed, and there was no selection of incoming patients, and evaluation and treatment were aimed at helping patients with reported problems. No attempts to conduct a research trial have been made. One limitation of this approach is that while a wide range of misophonia severity was observed, no subjects with very low levels of misophonia were present, which limits the extension of our observation to the general population of subjects with misophonia.

### 1.2. History of developing the concept of misophonia

It has been recognized for a long time that some people have a problem with tolerating sounds and exhibit negative reactions to ordinary sounds that do not evoke such reactions in an average listener. Various terms have been used to describe this condition, such as hyperacusis (Vernon and Press, 1998), recruitment, and decreased sound tolerance (DST), with hyperacusis being used most frequently (Baguley and McFerran, 2010; Jastreboff and Jastreboff, 2014b; Henry et al., 2022). This phenomenon may be related to various medical problems (e.g., migraine, autism, and Williams syndrome) (Van Borsel et al., 1997; Anari et al., 1999; Jastreboff and Jastreboff, 2001b, 2002, 2014b, 2018; Jastreboff and Hazell, 2004; Levitin et al., 2005; Formby, 2007; Formby et al., 2007; Hawley et al., 2007; Sheldrake et al., 2015; Pires et al., 2021), or it may affect a person without any identifiable etiology.

The definition of any disorder should be independent of its etiology; it should be specific, selective, and sensitive. These principles are generally recognized, and the term “idiopathic” (i.e., unknown) is used for disorders where the cause and origin have no known explanation.

Consequently, we proposed “to define DST as present when a subject exhibits negative reactions following exposure to sound that would not evoke the same response in an average listener” (Jastreboff and Jastreboff, 2002, 2014b). In this article, the above definition is used.

Until 2001, two forms of DST have been commonly recognized: hyperacusis and phonophobia. Hyperacusis is considered an auditory disorder, diagnosed, and treated by otolaryngologists and audiologists. It is defined in ICD-10 (code H93.23) as “*an abnormally disproportionate increase in the sensation of loudness in response to auditory stimuli of normal volume. Cochlear diseases, vestibulocochlear nerve diseases, facial nerve diseases, stapes surgery, and other disorders may be associated with this condition*” (https://www.icd10data.com/).

Phonophobia is considered to be either an auditory disorder or, typically, a psychological disorder—phobic anxiety disorder or specific phobia (code ICD-10-CM Diagnosis Code F40.298). “*Phonophobia is defined as a persistent, abnormal, and unwarranted fear of sound.”* It falls into the category of phobic anxiety disorders (code ICD-10-CM F40-F48) characterized by “*a strong, irrational fear of something that poses little or no actual danger”* (https://www.icd10data.com/). Hyperacusis has been and is still predominantly treated by audiologists and otolaryngologists. Phonophobia is treated mainly by psychologists but sometimes by psychiatrists and occupational therapists.

For over a decade (1990–2001), we have diagnosed and treated over 800 patients with DST at the University of Maryland at Baltimore and then at Emory Tinnitus and Hyperacusis Center, Atlanta, Georgia. All patients underwent comprehensive medical (performed by an otolaryngologist) and specific audiological evaluations. The evaluation and treatment of patients have been homogeneous, following the protocols of tinnitus retraining therapy (TRT) (Jastreboff and Hazell, 2004). No preselection of patients was done, and all patients with tinnitus and/or DST were seen, evaluated, and treated. As the center was mainly advertised as a tinnitus center, nearly all patients had bothersome tinnitus. Nevertheless, 66% of them reported problems with DST (Jastreboff and Jastreboff, 2002).

Results from 149 consecutive patients seen at the University of Maryland and Emory Tinnitus and Hyperacusis Center confirmed a high prevalence of DST in patients with tinnitus (Jastreboff and Jastreboff, 2002). In the discussed population of patients, 57.0% were diagnosed with misophonia and 29.7% with hyperacusis (Jastreboff and Jastreboff, 2002).

Accumulated observations pointed out that the majority of patients with DST did not fit into the definition of hyperacusis or phonophobia. These patients showed negative reactions to specific individual patient sounds while being able to tolerate other even much louder sounds which precluded classifying them as having hyperacusis. There was nothing particular or specific about the sounds themselves. “Specific/particular” is directed to the relation of “a sound” to “a patient,” i.e., bothersome sounds are particular for a given patient. Examples of a broad variety of reported sounds are presented in Table 1. These patients have not experienced fear, which precluded classifying them as having phonophobia.

**Table 1:** Sounds reported by our patients as evoking negative reactions.

Street sounds
Slamming doors
Sudden sounds
Leaf blowers
Lawnmowers
Swimming pool pump
Cafeterias/food courts
TV or radio with the volume set by a family member with normal hearing
Other people singing/humming
Vacuum cleaner
Boiling water
Sound of a refrigerator
Popping popcorn
Supermarket
Supermarket freezer
Grocery stores
Shopping malls
Crinkly bags
Crumpling or wrinkling paper
Hum of a computer
Hum of electricity
Sound of heating radiators
Office sounds (typing on a keyboard, printers, copy machine, and fax)
School breaks, cafeterias
Low-flying airplanes
Sound from other people's headphones
Laughter
Sniffing
Snoring
Chewing gum
Other people breathing
Lip-smacking
Sounds of eating
Swallowing
Chewing
Crunching sound
Clipping and filing fingernails
Toothbrush
Electric shaver
Hair dryer
Flushing toilet
Keys rattling
Moving hand on a surface
Sound of drawing with a felt-tipped pen
Dogs barking from the distance
Cat walking on a hardwood floor
Cat purring
Hamster on the wheel

Interestingly, sounds of nature, such as bird songs, running water, wind, and rain, are rarely reported as negative (Hazell et al., 2002). Modified from Jastreboff and Jastreboff (2014b).

To create a name for this newly recognized disorder, we asked an expert in the Greek language to provide us with a list of prefixes indicating something negative, which could be added to the word “phonia” (meaning voice, sound) to create a term labeling aforementioned patients. Specifically, in the situation when a subject exhibits negative reactions to particular for her/his patterns of sound, with acoustical strength (energy) of sound being irrelevant or of secondary importance. From the list, we selected the prefix “miso” which means “hate” in Greek.

Translating “misophonia” literally as “hatred of sound” is incorrect as is translating “chromatography” to “color drawing!” Regretfully, some professionals used this literal translation and started to promote the idea that a characteristic feature of misophonia is the hatred of sound. It was never our intention, and we have never used misophonia in this literal manner.

Similarities and differences between patients with hyperacusis vs. patients with misophonia suggested which characteristics should be used to identify patients with misophonia and differentiate them from patients with hyperacusis and patients with other medical disorders. Findings described below are the same as subsequent observations of our patients seen at Emory (after 2001), other audiological practices, and at the clinic of JHDF, Inc. (results in preparation). Our patients represented the whole range of severity of hyperacusis or misophonia from mild to very severe. All patients have sufficiently bothersome misophonia or hyperacusis to ask for help.

Interestingly, reports in the literature indicate that misophonia is present in William's syndrome and not hyperacusis, e.g., “a very striking characteristic is the hyperacusis or over-sensitivity to particular sounds” (Van Borsel et al., 1997) and “among people with WS, we found relatively few reports of true hyperacusis (the lowered threshold for soft sounds) or auditory fascinations/fixations, whereas 80% reported fearfulness to idiosyncratically particular sounds” (Levitin et al., 2005).

Some of our patients have autism, and the evaluation of their DST strongly supports that they have misophonia and not hyperacusis (Aazh et al., 2014; Jastreboff and Jastreboff, 2014a).

## 2. Diagnosis

The insightful analysis of various subcategories of decreased sound tolerance, including hyperacusis and misophonia, has been recently published (Henry et al., 2022). In defining misophonia and differentiating misophonia from other disorders, it is crucial to identify both its unique attributes, as well as features shared with other disorders, particularly common with hyperacusis. Misophonic triggers cover a wide variety of sounds with different spectral energy and without the indication of the preferred range of frequencies or the range of sound energy (Jastreboff and Jastreboff, 2014b; Jager et al., 2020; Hansen et al., 2021; Vitoratou et al., 2021; Henry et al., 2022). It is important to recognize that the physical strength of misophonic triggers—their loudness—may play some role, but only secondary. This reflects the general principle that a stronger stimulus evokes a stronger and/or faster reaction (Palmer et al., 2005; Causer et al., 2013). The reaction to a misophonic trigger is only weakly related to its strength; nevertheless, the trigger of higher intensity will tend to evoke a stronger reaction due to this general principle. This dependence is, however, a dominant feature in hyperacusis.

Notably, specific patterns and meanings of sound are commonly observed in misophonia, e.g., sounds created by humans (eating, breathing, and voices) which may have different frequency ranges and intensities but have similar meanings. This is in strong contrast with hyperacusis, where the intensity of the sound plays a crucial role (Jastreboff and Jastreboff, 2014b; Henry et al., 2022). In addition, one of the characteristic features of hyperacusis is the negative reactions to high-pitch sounds (Jastreboff and Jastreboff, 2014b; Sheldrake et al., 2015), disregarding how and where they are produced. The pattern and meaning of sound do not play any clear, significant role.

Negative reactions to sounds among patients with hyperacusis and misophonia are similar and frequently identical (Table 2). Importantly, many of these reactions were the same as in patients with tinnitus or other chronic medical disorders (e.g., back pain, cancer, and general sensory over-sensitivity) (Jastreboff and Jastreboff, 2014b). Thus, reactions to sound cannot be used exclusively as a characterizing/discriminating factor for identifying patients with misophonia and for separating patients with misophonia from patients with hyperacusis.

**Table 2:** Negative reactions frequently reported by patients with misophonia or hyperacusis.

Irritation
Annoyance
Tension
Frustration
Urge to escape (run)
Urge to cry
Feeling of physical pain
Feeling of being restrained in doing things
Feeling uncomfortable (discomfort)
Inability to concentrate
Inability to enjoy activities/events/situations, particularly involving louder or specific sounds
Increased awareness of sounds (being forced to monitor sounds)
Fear of sounds
Emotional distress
Uneasiness
Worry
Anger
Stress
Being argumentative
Becoming aggressive
Decreased ability to control own reactions
Disgust
Sadness
Anticipation and the need to monitor/control the surroundings (being on the look-out)
Apprehension
Distraction
Continuous alertness

Modified from Jastreboff and Jastreboff (2014b).

Our observations showed that factors related to the type of reactions (e.g., patient's psychological profile or rarely observed presence of psychiatric disorders) were of no significance for discrimination between patients with misophonia and hyperacusis or patients with tinnitus. The main distinguishing factor was the discrepancy between acoustical features (energy) carried by a sound and the extent of negative reactions observed in patients with misophonia. Furthermore, there is a dependence of reactions on the person who is generating a bothersome sound and the environment where that sound is presented. In contrast, in hyperacusis, there is a positive relationship between sound's energy and the extent of negative reactions; the meaning of a sound will be irrelevant. If the presence and extent of negative reactions depend on who is generating the sound and the environment in which it is produced, it excludes hyperacusis.

Importantly, the audiological evaluation does not allow for discrimination between misophonia and hyperacusis as lower than normal values of loudness discomfort levels (LDLs) can exist in both disorders. While low (below 90 dB HL) LDLs values are needed for the diagnosis of hyperacusis, they are not a characteristic feature of misophonia. It has been shown that LDL values and patients' ratings of decreased sound tolerance are poorly correlated (Anari et al., 1999; Jastreboff and Jastreboff, 2014b, 2018; Zaugg et al., 2016; Henry et al., 2022). There is a building consensus that LDL should not be used to diagnose the presence of hyperacusis or misophonia and that specific, detailed questionnaires are necessary. Therefore, a detailed interview aimed at finding discrepancies between the acoustical characterization of bothersome and not bothersome sounds is essential. Unfortunately, there are no generally accepted, validated questionnaires, neither for hyperacusis nor misophonia (Henry et al., 2022).

Additional help in the diagnosis of misophonia is provided by comparing the shape of the audiogram and the shape of LDL curves, which typically exhibit parallelism of shapes between the audiogram and LDLs for misophonia. There is a lack of this relation for hyperacusis (Jastreboff and Hazell, 2004). A detailed description of diagnostic protocols is currently in preparation.

The characteristics described above delineate the situation of pure misophonia and pure hyperacusis. Clinical observations show that misophonia and hyperacusis frequently coexist (Jastreboff and Jastreboff, 2002, 2014b) but need to be treated concurrently (Jastreboff and Jastreboff, 2012, 2014b).

## 3. Current status in the field of misophonia

Research, both basic (oriented toward delineating mechanisms of misophonia) and aimed at searching for effective treatments for this disorder, is strongly hindered by a lack of consensus on the definition of misophonia (Swedo et al., 2022). Consequently, there is a lack of an established, validated method for identifying the presence, diagnosis, and assessment of misophonia severity. Currently, since various groups use different rules to identify patients with misophonia based on different definitions (and various postulated etiologies), it is difficult to combine research and clinical data as subjects studied/treated by different centers do not represent the homogenous population of patients with misophonia but, rather, various patient sub-populations and “comparisons between study cohorts are not possible” (Swedo et al., 2022).

Importantly, the results of reported studies are corrupted by the lack of exclusion from the evaluated group of subjects with hyperacusis. This is a significant issue because while reactions of hyperacusis and misophonia to bothersome sounds are similar (even identical), clinical results show that treatment effective for hyperacusis is not working for misophonia (Jastreboff and Jastreboff, 2012, 2014b), indicating different mechanisms of these two disorders. It has been observed that hyperacusis is seen in patients with misophonia (Henry et al., 2002; Jastreboff and Jastreboff, 2002; Swedo et al., 2022) and that “for any given individual, the symptoms of misophonia should not be better explained by any co-occurring disorders” (Henry et al., 2002). Since in hyperacusis, symptoms are very similar or even identical to that observed in misophonia, therefore, for research and treatment, patients with hyperacusis should be excluded and investigated separately from patients with misophonia only.

The danger of ignoring this problem has been clearly demonstrated with tinnitus when an article published by a highly respectable group presenting the results of an imaging study postulating changes related to the presence of tinnitus turned out to describe the effects of hyperacusis present in some of the studied subjects, the existence of which was not considered (Melcher et al., 2000). Subsequently, these results have been withdrawn after an overlooked contribution of hyperacusis has been taken into account (Melcher et al., 2009; Gu et al., 2010).

The situation is further complicated by a push toward determining presumed etiology before establishing a definition. To create a definition of a disorder, it is not necessary to know its etiology, and therefore, the term “idiopathic” is used in many cases (e.g., idiopathic intracranial hypertension, idiopathic ventricular tachycardia, idiopathic sudden sensorineural hearing loss, and Ménière's disease) (Altemose and Buxton, 1999; Ciccone et al., 2018; Rehder, 2020; Desiato et al., 2021; Dai et al., 2022; de Cates and Winters, 2022; Marchioni et al., 2022).

Proposing a specific etiology before constructing a definition is premature and may have a detrimental effect. Unfortunately, this danger is clearly visible in misophonia when professionals from various fields impose criteria for identifying misophonic subjects based on presumed etiology. Currently, there are two, contrasting approaches to misophonia as highlighted in Swedo's study (Swedo et al., 2022): first, “medical” (Jastreboff and Jastreboff, 2002, 2015; Edelstein et al., 2013; Cavanna and Seri, 2015) and second, “psychiatric” (Schroder et al., 2013). The consensus committee concluded that at the moment, there is no sufficient evidence to select one of these approaches over the other, but “that underlying organic etiology of the disorder cannot be ruled out” (Swedo et al., 2022).

Such a situation creates an additional problem—specialists from classical medical fields (e.g., otolaryngologists) assess the severity of the problem using characteristic features of the disease (e.g., as for evaluating tinnitus severity), while professionals from mental health fields tend to use questionnaires aimed at reactions evoked by disease and its impact on life.

A classic example of the detrimental effect of imposing unproven etiology is the history of Ménière's disease. It was proposed over 40 years ago that the etiology of Ménière's disease (symptoms: vertigo, nausea, loss of hearing, tinnitus, and loss of balance) is increased pressure of the endolymph in the inner ear (“endolymphatic hydrops”). Since then, practically, all animal research and clinical treatments, including popular endolymphatic shunt surgery, were based on this concept. Recent studies, including clinical blind studies, have shown that Meniere's disease should not be based on the endolymphatic hydrops theory (Thomsen et al., 1981a,b, 1998; Bretlau et al., 1984; Merchant et al., 2005; Devantier et al., 2019). Unfortunately, because of the postulated incorrect etiology, decades of research became useless, and many patients underwent serious operations which were not better than placebo.

## 4. Definition of hyperacusis and misophonia based on observations of patients with DST, without postulating any specific etiology

Based on the observations described above, the following definition of misophonia and hyperacusis has been proposed in 2001 (Jastreboff and Jastreboff, 2000, 2001b) and has been reiterated in 2014 (Jastreboff and Jastreboff, 2014b).

*Hyperacusis is defined as present “when negative reactions to a sound depend only on its physical characteristics (i.e., its spectrum and intensity). The sound's meaning and the context in which it occurs are irrelevant.” “For example, a patient will react identically to the sound of a knife hitting china in any situation or setting. This individual also will react negatively to all other high-intensity sounds*.” (Jastreboff and Jastreboff, 2014b).

“*Misophonia is present when an abnormally strong reaction occurs to a sound with a specific pattern and/or meaning to an individual. The reaction may depend on the environment where the offensive sound is presented. The physical characteristics of the sound are secondary. Indeed, the strength of the misophonic patient's reaction is only partially determined by the sound's physical characteristics. Frequently, a person with misophonia will respond strongly to a soft sound of a specific pattern (e.g., a voice, the sounds of eating) but not react to other, much louder sounds (e.g., loud music). Furthermore, the individual may react to a given sound in one setting (such as in his or her home) but not react to the same sound in another setting (such as in the home of a friend). The patient's negative reaction to the sound depends on nonauditory factors such as his or her previous evaluation of the sound on the belief that the sound is a potential threat or that exposure to it will be harmful. The sound may be associated with a previous negative experience. The patient's psychological profile and the context in which the sound occurs are important as well*.” (Jastreboff and Jastreboff, 2014b).

It is proposed that DST is a summation of the effects of misophonia and hyperacusis. No other conditions or restrictions other than the ones listed above were imposed on deciding whether a patient has misophonia or hyperacusis. In this approach, phonophobia is considered a specific case of misophonia when fear is the dominant emotion.

Unfortunately, the definition of misophonia proposed in Swedo et al.'s study describing the Delphi method (Swedo et al., 2022) is insufficiently specific and selective and does not discriminate misophonia from hyperacusis.

## 5. Reasoning leading to the neurophysiological model of misophonia and hyperacusis

While proposed behaviorally based definitions do not assume any specific etiology, it is possible to speculate which physiological mechanisms are involved and responsible for these two phenomena based on the observed features of patients with misophonia and hyperacusis. Discussion yielding a proposed model and presumed mechanisms of hyperacusis and misophonia have been published in detail already (Jastreboff and Jastreboff, 2014b, 2018), and only the main points are presented here.

In hyperacusis, the dependence of the presence and strength of negative reactions on the acoustical characterization of bothersome sounds highlights that a crucial part of the mechanism of hyperacusis is within auditory pathways. Furthermore, the irrelevance of the meaning of bothersome sounds and the high level of repeatability of reactions indicates that the subconscious part of the auditory pathways plays a dominant role. The term “subconscious” is used to denote part of the brain which is outside of the conscious control of a person. Responses evoked by this part of the brain are automatic, involuntary, fast, and governed by principles of conditioned reflexes.

**Figure 1:**
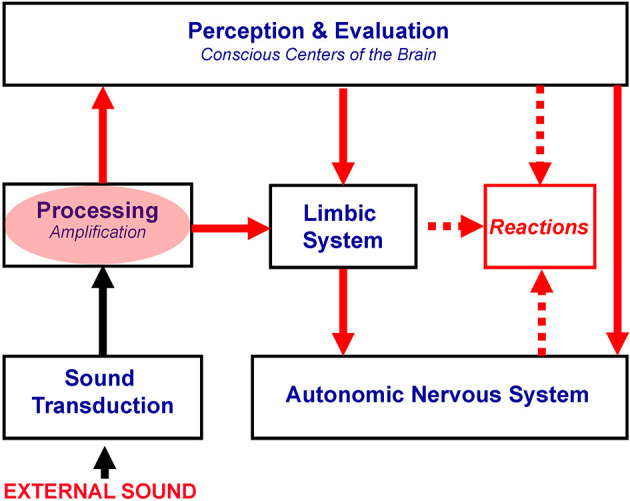
Proposed mechanisms of hyperacusis. The red oval marks subconscious centers of the auditory pathway with increased gain, resulting in the over-amplification of neuronal activity evoked by bothersome sounds. This over-amplification is postulated to be responsible for hyperacusis. Red arrows show the spread of the enhanced neuronal activity, yielding the overactivation of the limbic and autonomic nervous systems, which are dominant in the generation of negative reactions.

Therefore, hyperacusis reflects an abnormally strong reactivity of the subconscious part of the auditory pathways to sound, which in turn yields the activation of the limbic and autonomic nervous systems (Figure 1). Notably, proposed mechanisms of hyperacusis have been incorporated from the beginning in the neurophysiological model of tinnitus and in TRT, with hyperacusis being crucial for patients' classification and treatment (Jastreboff and Hazell, 1993, 2004; Jastreboff, 1995), but for brevity, “hyperacusis” was not included in the title.

In the case of misophonia, the irrelevance of acoustic characterization of misophonic triggers shows that the auditory system plays a secondary role and works typically within the norms. The association of certain sounds with strong reactions plays a dominant role and indicates that functional connections between the auditory, limbic, and autonomic nervous systems are enhanced for specific patterns of sound (Jastreboff and Hazell, 2004; Jastreboff and Jastreboff, 2004, 2013).

There is a broad variety of misophonic triggers described in the literature (Jastreboff and Jastreboff, 2014b; Jager et al., 2020; Hansen et al., 2021; Vitoratou et al., 2021). It is postulated that the dominant feature of misophonic triggers is their meaning and the association of a misophonic trigger with a subject's past experience (including an association with a specific person, place, or situation), and what is in our model explained by invoking the concept of complex conditioned stimuli. This concept shifts the focus from a single, physical stimulus to a complex one involving other dimensions, e.g., other elements of a sensory scene which includes a misophonic trigger as a part of the scene, personal relations to the person generating sounds, and the ability to control the environment (Jastreboff and Jastreboff, 2014b). Furthermore, the concept of complex conditioned stimuli can explain why certain classes of sound have a higher probability of being misophonic triggers, or why typically sounds made by members of a close family are more bothersome than the same sound produced by strangers. It also explains the observation that when patients with misophonia do not attribute a sound of misophonic triggers to its original source, it generates lower levels of negative reactions—if the physical characterization of the misophonic triggers alone was a determining factor, the reactions would be the same. We are utilizing this concept in one of the protocols for misophonia treatment [protocol (4); its basis is outlined in Jastreboff and Jastreboff (2014b)].

**Figure 2:**
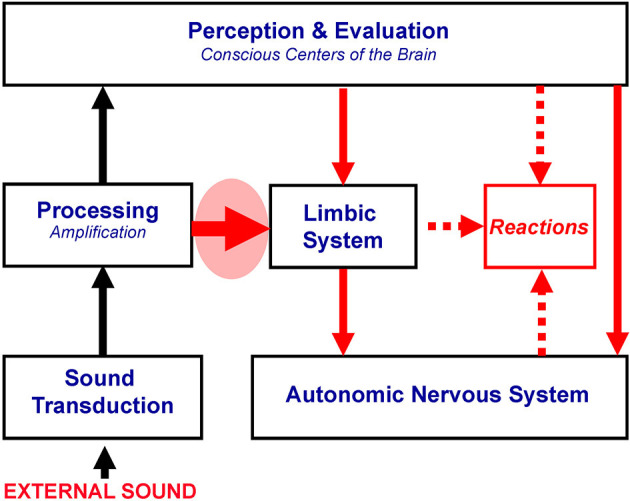
Proposed mechanisms of misophonia. The thick red arrow in the red oval marks the functional linking of the subconscious part of the auditory system with the limbic and autonomic nervous systems, postulated to be responsible for misophonia. All systems in the brain could be working within the norm. Other symbols are described in Figure 1.

Connections between the auditory and the limbic and autonomic nervous systems involve both the conscious, cognitive part of the brain, and the subconscious paths, with the subconscious paths governed by the principle of conditioned reflexes. The observation that most patients realize that misophonic triggers are not dangerous *per se* and that their reactions are disproportionate to the acoustical characteristics of these sounds and their meaning suggests that conscious analysis plays a secondary role. Furthermore, the response to misophonic triggers is fast, supporting a dominant role of the subconscious connections and the lack of the need for conscious analysis and evaluation of these sounds (Jastreboff, 2008; Jastreboff and Jastreboff, 2018). As a result, even if a person fully understands that a given sound is not dangerous or threatening, strong negative reactions are still evoked. Mechanisms postulated for misophonia are presented in Figure 2.

Proposed mechanisms of misophonia are supported by the results of physiological investigations by Edelstein et al. (2013). The authors found experimental evidence that misophonia produces distinct autonomic effects and suggested that mechanisms of misophonia involve aberrant functional connections between the auditory and limbic systems. Interestingly, pain reported by some patients with misophonia can be created by an overactivated autonomic nervous system, which causes the activation of the tensor tympani muscle (Jastreboff, 2010; Jastreboff and Jastreboff, 2013), yielding tensor tympani syndrome (e.g., fullness, pulsation, and ear pain) (Klochoff, 1979). Tensor tympani syndrome is frequently observed in patients with misophonia, and the treatment of misophonia results in its elimination (Jastreboff and Jastreboff, 2013, 2014b, 2018).

In summary, misophonia reflects abnormally strong reactions of the autonomic and limbic systems resulting from enhanced functional connections between the auditory, limbic, and autonomic systems for particular for a given patient patterns of sound. It should be noted that there is nothing particular or specific about the sounds themselves, and any sound can become a bothersome misophonic trigger. “Specific/particular” is directed to the relation of “a sound” to “a patient,” i.e., bothersome sounds are particular to a given patient.

In the proposed mechanisms of DST, it is postulated that in both misophonia and hyperacusis, the subconscious brain plays a dominant role, and for misophonia, the subconscious conditioned reflexes are of crucial importance.

Note that in the proposed neurophysiological model of DST, other systems in the brain are not excluded and may play a role. However, applying Ocam's razor principle, the simplest explanation which is sufficient to explain observed phenomena should be used. Therefore, only the limbic and autonomic nervous systems, which we believe cannot be excluded and which play a crucial role, are highlighted in the model.

Other brain systems could be included and should be considered while investigating mechanisms of misophonia (Kumar et al., 2017, 2021; Brout et al., 2018; Schroder et al., 2019).

## 6. Proposed treatments of misophonia and hyperacusis based on the model and its results

### 6.1. Reasoning yielding proposed treatment

The delineated neurophysiological model creates a basis for proposed mechanism-based treatments for hyperacusis and misophonia (Jastreboff and Jastreboff, 2014b, 2018). Both treatments follow the principles of TRT and involve counseling and sound therapy. Despite similarities, the implementation of counseling and sound therapy differs substantially between these two disorders. Our clinical experience shows that treatment which is effective for hyperacusis is not helpful for misophonia (Jastreboff and Jastreboff, 2012, 2014b).

Since in hyperacusis, it is postulated that the problem arises from an abnormal increase of gain within subconscious auditory pathways, treatment aims at decreasing this gain utilizing desensitization procedures. Therefore, counseling and sound therapy focus on mechanisms of general desensitization to sound provided by constant, 24/7 exposure to neutral sound, and the general enrichment of environmental sounds.

Treatment for misophonia differs from hyperacusis significantly and involves additional mechanisms not utilized for hyperacusis. As the problem arises from the creation of subconscious functional connections governed by principles of conditioned reflexes, both counseling and sound therapy work together to eliminate (or substantially weaken) these functional connections. They are expanded to include mechanisms and principles of active extinction of these conditioned reflexes, with stress on principles of generalization of stimuli and complex conditioned stimuli (Jastreboff and Jastreboff, 2014b). Sound therapy (while still implementing weakening neural representation of misophonic triggers by neutral sounds) focuses on creating a positive association to sound in general and on decreasing/removing negative reactions to misophonic triggers by purposefully creating and then modifying complex conditioned stimuli, which include misophonic triggers. Four classes of protocols with sound utilizations have been described (Jastreboff and Jastreboff, 2014b).

In most cases, misophonia and hyperacusis occur together wherein both of them need to be treated concurrently. As the treatment of misophonia is more complex, it requires more extensive counseling and takes more time than the treatment of hyperacusis (Jastreboff and Jastreboff, 2014b).

### 6.2. Overview of the clinical effectiveness of TRT

A total of 201 consecutive patients diagnosed with DST were treated with TRT (Jastreboff and Jastreboff, 2014b). All the patients underwent detailed audiological and medical clinical evaluation and treatment as described previously (Jastreboff and Jastreboff, 2000, 2003, 2014b; Henry et al., 2002).

A total of 184 (91.5%) patients exhibited misophonia with or without hyperacusis; of which, 17 patients (8.5%) had hyperacusis alone, and 56 patients (27.9%) had misophonia only. Detailed initial and follow-up interviews have been conducted with the help of structured interview forms (Jastreboff and Jastreboff, 1999; Henry et al., 2003). The criteria for evaluating patients' DST and treatment outcomes have been presented elsewhere (Jastreboff and Jastreboff, 2014b). The duration of all treatments (for misophonia, hyperacusis, and tinnitus) was set to be at least 9 months even if the patient showed clinically significant improvement in a shorter time. On average, patients exhibited a noticeable improvement after 3 months. The improvement progresses gradually over time, without the indication of saturation, even after reaching the level of clinically significant improvement.

A total of 165 out of 201 patients with DST showed a significant improvement (success rate of 82.1%). For misophonia with or without hyperacusis, 152 out of 184 patients showed a significant improvement (success rate of 82.6%) (Jastreboff and Jastreboff, 2014b). In some cases, both for misophonia and hyperacusis, it was possible to completely eliminate the problem (Jastreboff and Jastreboff, 2014b). Relapse is very infrequent based on the observation of patients over a period of up to 20 years (Jastreboff and Jastreboff, 2018).

## 7. Comments on other proposed approaches to misophonia and the issue of misophonia etiology

While for the definition of misophonia, the etiology and potential mechanisms involved in this phenomenon are irrelevant; however, based on postulated mechanisms, it is easier to conduct a research study of this disorder and propose a mechanism-based treatment. From this perspective, the question “Does misophonia belong to a field of otolaryngology, audiology, neurophysiology, neurology, psychology, or psychiatry?” is significant. A thoughtful analysis of the potential mechanisms of misophonia was presented by Palumbo et al. (2018).

The definition of misophonia has been proposed in 2001 (Jastreboff and Jastreboff, 2001a,b). In 2013, an article was published where misophonia was redefined and classified as a psychiatric disorder (Schroder et al., 2013). The authors proposed six stages of developing misophonia, based on the observation of 42 patients in their psychiatric center. These stages were considered to define misophonia: (1) human-made sounds create anger, (2) leads to a deep sense of loss of self-control, (3) anger is recognized as excessive, (4) misophonic triggers are avoided; otherwise, it results in intense discomfort and anger, (5) anger, disgust, and avoidance cause significant distress and interference with everyday life, and (6) this process cannot be explained by psychiatric disorders such as OCD or PTSD. Based on these criteria, a questionnaire to assess the presence and severity of misophonia has been proposed—Amsterdam Misophonia Scale (A-MISO-S) (Schroder et al., 2013).

Our observation of over 800 patients with DST treated from 1990 to 2001 was in clear disagreement with those described by Schroder et al.: (1) Misophonic triggers are not exclusive sounds produced by humans, and while anger is sometimes present, it exists only in a portion of patients with misophonia; (2) some patients feel a loss of self-control, but aggressive outbursts happen in only a few patients, particularly the very young; (3) anger is not present in the majority of patients, and many patients believe that other people are behaving in an unreasonable/disrespectful manner, and their feelings/reactions are normal and justified; (4) avoidance is observed and is the same as in patients with hyperacusis; while indeed some reactions may be of intense discomfort, disgust, or occasionally anger, however, reactions frequently involve just annoyance or some degree of discomfort; and (5) negative reactions can be mild and not necessarily strong, and they do not have to include anger or disgust; they are the same for patients with tinnitus or hyperacusis.

Criteria proposed by Schroder to define and characterize misophonia are neither specific nor selective. Using these criteria, several other health problems (e.g., hyperacusis, tinnitus) could be classified as misophonia. In our opinion, the problem is that Schroeder's and other similar definitions are focused exclusively on dissecting reactions of patients with misophonia (which are not unique to misophonia) and are not addressing characteristic properties of misophonia, i.e., negative reactions are evoked by particular for a given patient's patterns of sound, with the occurrence and strength of reactions typically depending on a source and the environment in which the patient is exposed to misophonic triggers. Therefore, it seems that the definition proposed by Schroder et al. (2013) describes a subset of patients with misophonia and should not be applied to the general population of patients with misophonia.

There are additional observations arguing against the classification of misophonia as a psychiatric or psychological disorder. All patients based on which the concept of misophonia was proposed have been thoughtfully evaluated by otolaryngologists (45-min detailed, comprehensive medical evaluation), who were taking into account the potential comorbidity of psychological/psychiatric disorders and were ready to make proper referrals if needed. Extra attention has been paid to the presence of psychological or psychiatric problems as tinnitus has been anecdotally reported to lead to suicide.

Notably, it appears that the vast majority of our patients did not have any obvious psychological or psychiatric disorder. Indeed, while we have a few patients diagnosed with bipolar disorder, high levels of OCD, pre-existing depression, etc. (with or without misophonia), we encounter only a small proportion of patients with psychiatric or psychological problems requiring specialized attention. It should be noted that anxiety and depression are frequently encountered in patients and typically have been created or significantly enhanced after the emergence of tinnitus or DST, and therefore, patients were diagnosed with “situation-evoked” anxiety or depression. A vast majority of patients did not require specialized psychological/psychiatric treatment related to tinnitus, hyperacusis, or misophonia. Thus, while misophonia can exist in patients with psychiatric/psychological disorders, the presence of these disorders does not appear to be directly linked to misophonia. This argues against the postulate that misophonia or hyperacusis has a psychiatric basis and should be considered a psychiatric or psychological disorder.

Observations of lacking psychological/psychiatric differences between patients with misophonia, hyperacusis, and tinnitus have also been reported by Erfanian et al. (2019). Based on their results, they concluded that “*Similar to misophonia, patients with tinnitus and hyperacusis tend to show abnormal scores on psychological assessment, indicating that they experience a high level of co-morbidity with symptoms of psychiatric disorders [33].”* Furthermore, they highlighted the physiological mechanisms of situation-evoked depression: “*However, depressive symptoms are the possible consequences of not only misophonia but also similar disorders such as hyperacusis and tinnitus [35]. Hence, the co-occurrence of depressive symptoms in misophonia can be explained by the activation of the survival reflex which declines the ability of a subject to enjoy daily activities [35]. Having said that, we do not suggest that depressive symptoms explain the misophonic symptoms, while the majority of our patients do not meet with[sic] the clinical criteria of depressive disorders (as also suggested by [2,3])*.” (Erfanian et al., 2019).

The additional argument is that the TRT treatment of patients with misophonia yields 83% effectiveness, which to our knowledge is higher than other published results. This is based on the neurophysiological model of tinnitus and DST which does not involve a postulate of psychological or psychiatric mechanisms and does not use tools for treatment from the fields of mental disorders. Specifically, in a study presenting results of CBT for misophonia (Schroder et al., 2017) out of 90 patients, 48% showed improvement. In Jager et al. (2020) study evaluating the effectiveness of CBT in a randomized clinical trial, the authors used several scales and reported that 37% of their 54 patients showed statistical improvement (Jager et al., 2020). It is important to note that Jarger's study is so far the only publication that presents results of a randomized clinical trial (RCT) for misophonia while our results represent a Level IV of evidence (Series of Cases) and as such are not as strong as RCT (Level II) (Poehling, 2004; Burns et al., 2011; El-Gilany, 2018).

## 8. Discussion

In this study, the following topics are discussed: (1) definitions, (2) neurophysiological model of DST (both misophonia and hyperacusis) highlighting potential shortcomings arising from the current status of development in the field of misophonia, (3) critical assessment of the results of TRT treatment, and 4) lines of future works.

### 8.1. Definition of misophonia

As argued in Section 3, the lack of agreement on the cause(s), origin, or mechanism(s) of misophonia should be irrelevant to the proposed definition. Etiology, while helpful, is not crucial even for research or development of treatment methods. In fact, incorrect etiology can hinder the research and development of treatment.

The definition presented in this issue (Swedo et al., 2022), which represents the results of using the Delphi method to reach an expert agreement, is a very important step in the field of misophonia and creates the basis for further work toward refining the definition of misophonia, which would reach a consensus of professionals working in the field. “*The Delphi method works on the assumption that group judgments are more valid than individual ones. The approach is an effective iterative process with repeated rounds of evidence evaluation and voting to determine a consensus among a group of experts with different knowledge and varying levels of expertise about a particular topic”* (Swedo et al., 2022). Importantly, Swedo et al. made it clear that they do not attempt to postulate any specific etiology for misophonia (Swedo et al., 2022). Unfortunately, there are some points that raise concerns.

The creation of the definition seems to be biased toward delineating reactions, which are however not unique to misophonia. The characteristic feature of misophonia, i.e., reactions to complex stimuli, with the auditory component being important, but only one of the sensory dimensions involved in creating a misophonic trigger, has been underplayed. Whether a given sound is a misophonic trigger is highly dependent on patients' previous experience. In other words, patients with misophonia react to complex conditioned stimuli with their reactions dependent on their previous encounters with these sounds and not determined by acoustical characterization of misophonic triggers. Consequently, the definition of misophonia proposed in Swedo et al.'s (2022) study is not sufficiently specific and selective, and it does not discriminate misophonia from hyperacusis. Thus, it does not provide clear guidance for excluding subjects with hyperacusis while conducting misophonia research, treating patients, and creating new treatments specifically tailored to misophonia.

In the Delphi method, the composition of a committee is crucial (Swedo et al., 2022). Correcting the under-representation of professionals who are working with patients with misophonia on an everyday basis at a purely clinical level would be beneficial. This is evident while exploring the work and publications of the authors of Swedo et al.'s (2022) study.

All members of the committee were respected professionals in their fields. However, the majority of the committee's members deal with misophonia subjects in a research-oriented environment, with the selection of participants who classify for their definition of misophonia, or members who had limited clinical experience with patients with misophonia. Out of 15 voting members, over 50% have none or only one prior publication on misophonia; 80% had no publication, having the term “hyperacusis” in the title or abstract; 67% were psychologists or psychiatrists; only 33% had clinical experience with misophonia; and only 20% had clinical audiology background. These factors had repercussions on the familiarity with hyperacusis and created a bias toward psychological/psychiatric approaches.

Combined with a tendency of focusing on the etiology of misophonia and on arguing which professional category should be involved in working and providing clinical services to patients with misophonia created certain biases toward fields of psychology and psychiatry while ignoring the importance of hyperacusis. Moreover, it has been pointed out that it may be preferable for the Delphi process to not only include researchers and clinicians but also people who have misophonia (Henry et al., 2022).

In our opinion, proposed in the 2002 definitions of DST, hyperacusis and misophonia (Jastreboff and Jastreboff, 2002, 2014b, 2018) seem to fulfill the requirements for optimal definitions of a medical disorder. These definitions do not imply any etiology; they are broad enough to encompass all subjects exhibiting the given disorder, while at the same time, they are selective and sensitive. It is of particular importance that they allow for separating patients with hyperacusis from those with misophonia despite observations that the reactions of these two groups of patients are very similar, if not identical.

### 8.2. Proposed model of DST

The described model of DST (misophonia and hyperacusis) is supported by patients' observations. Importantly, treatment based on this neurophysiological model of DST has a high success rate with persisting improvement and without relapse (Jastreboff and Jastreboff, 2014b).

It is proposed that misophonia is based on subconscious conditioned reflexes linking the auditory system with other systems in the brain, particularly with the limbic and autonomic nervous systems (Jastreboff and Hazell, 2004; Jastreboff and Jastreboff, 2014b, 2018). Misophonic reactions can be developed to any type of sound and in any person—it is enough that certain sound(s) appear in a situation of a high level of emotional distress when the subject experiences pain or other negative sensations (e.g., as a result of hyperacusis or tensor tympanic syndrome, annoyance, or anxiety) or resulting from the subject associating a sound with something negative (e.g., a belief that a specific sound enhances tinnitus, produces a hearing loss, or is produced by a person who is perceived in a negative manner).

As such, misophonia (while it may significantly affect patients' lives) is not a pathological or psychological/psychiatric disorder. Occasionally, patients benefit from additional psychological treatment to address issues like stress, family problems, or obsession, but they are not a required, necessary part of our treatment. These treatments can be used as an adjunct, when needed, and then patients are referred to proper professionals.

Indeed, misophonia can be induced in any person by creating an association of some specific patterns of sound with negative reinforcement (some real examples from our patients: the sound of steps of a stepmother who was purposefully following a teenage patient to make his life miserable; the sound of kissing made by a sibling to irritate a patient, accompanied by negative comments; the clicking sound made by the claw of a cat walking over hard surfaces, where this particular patient disliked cats jumping on a table). Detailed interviews with over 1,000 patients with misophonia, and the clinical cases provided here, fully support the proposed model based on the involvement of subconscious conditioned reflexes as they describe examples of classical Pavlovian conditioning with some sound (acting as the conditioned stimulus) present when a subject is in a negative emotional state (acting as the unconditioned stimulus). The psychological profile of a patient as well as psychological or psychiatric problems (e.g., OCD) may influence the likelihood of misophonia emergence.

### 8.3. Critical assessment of the results of TRT treatment

Results obtained from 201 consecutive patients with DST showed an over 80% success rate without relapse (Jastreboff and Jastreboff, 2014b) and provided additional support to the presented model. However, we realize the limitations of reported results: 1) the lack of a control group, 2) the assessment of improvement was done based on Likert scales of our initial and follow-up questionnaires, and 3) results were not collected in a clinical trial and belongs to category case series (Level IV of clinical validity) (Poehling, 2004; Burns et al., 2011; El-Gilany, 2018).

Since the patients were not part of a clinical trial, there was no control group, e.g., “waiting list” as delaying treatment would be considered unethical. In reality, there was an unintentional “waiting list” as patients typically needed to wait at least a month to get into the treatment. There was no noticeable improvement noted during the period of waiting (observation based on the information provided in forms and questionnaires submitted by patients when they decided to enroll in the treatment).

Nevertheless, there are observations supporting the significance of the reported results: (1) cases were consecutive; (2) patients were treated in a uniform manner; (3) patients were followed for at least 2 years with multiple contacts at which the same structured interview was applied; (4) improvement had to be present on more than one scale; (5) some patients exhibited getting cured of misophonia and/or hyperacusis; (6) a substantial proportion of these patients who improved significantly with TRT treatment underwent unsuccessful treatment(s) by other professionals for several years (sometimes more than 10 years) for misophonia without improvement; and (7) results were highly statistically significant. These results cannot be explained by the placebo effect. Results from several hundred additional patients treated over subsequent years are in full agreement with those already reported (in preparation).

### 8.4. Proposed lines of future works

It is possible to suggest a certain sequence of investigations, which should be performed before going deeper into potential mechanisms of misophonia, and then work toward proposing new, mechanisms-based treatments. These investigations should allow for clarifying what misophonia is and what it is not.

The use of several, incompatible definitions of misophonia and various questionnaires, guided by different definitions, creates a situation where it is currently impossible to combine data from different studies and reach conclusions regarding the mechanisms of misophonia and its treatment. For example, if a questionnaire is based on an incorrect, literal interpretation of the name “misophonia” and the majority of questions involve “hate of sound,” this makes it invalid for the evaluation of misophonia as it only detects a specific subpopulation of patients with misophonia.

One of the crucial current problems is that reported data were collected from various sub-populations of patients who have misophonia with a bias created by an accepted definition of misophonia and its postulated etiology. Consequently, the selection of subjects in reported studies is not constant between published studies, and various particular groups of patients with misophonia were evaluated in a given study. Finally, in all these studies, subjects with hyperacusis have not been excluded and may incorrectly affect the results. This causes difficulty in deciding to what extent reported results are linked to misophonia.

We believe that it is necessary to first create a consensus on the definition based on clinical facts, unbiased by the theoretical model, without presuming the etiology of misophonia, and then create definition-based questionnaires for misophonia and hyperacusis. Only then it will be possible to indicate the potential etiology of misophonia and carry out works related to its mechanisms and treatments.

Considering the heterogeneity of patients evaluated by various groups, it is important to perform studies with an open acceptance of patients with DST, who exhibit negative reactions (of any kind) following exposure to sound that would not evoke the same response in an average listener. Next, subjects should be separated into hyperacusis and misophonia subgroups based on existing criteria for hyperacusis, and the results should be analyzed separately. An additional group of patients with tinnitus only should be recruited to provide a control group of patients who show similar emotional and autonomic reactions without exhibiting DST. These studies would allow for exploring the issue of comorbidity of other disorders, audiological description, and identifying characteristic features of the misophonic population.

The next set of studies could be focused on clarifying whether there are differences in patients' reactions and acoustic characterization of bothersome sounds by analyzing and comparing reactions to sounds reported by patients with misophonia to reactions reported by patients with hyperacusis.

A consensus is needed regarding the specificity of the reactions of the autonomic nervous system in evoking reactions observed in patients with misophonia. This can be clarified by performing studies oriented toward the analysis of the physiological manifestation of the excitation of the autonomic nervous system in patients with misophonia as compared with patients with hyperacusis and observed in patients with other chronic medical disorders. The expectation is that there will be no significant differences in autonomic reactions between patients who have only misophonia, or only hyperacusis, or other medical problems. If results confirm this prediction, then consequently, autonomic reactions cannot be used in the diagnosis of misophonia.

Knowledge of misophonia etiology is interesting, and the assessment of a potential psychological or psychiatric disorder is crucial for obtaining insight into this issue. The analysis of the prevalence of diagnosed psychological and psychiatric disorders in a group of subjects who have only misophonia, only hyperacusis, and only tinnitus, and comparing them with the prevalence observed in the population of subjects with chronic health problems are needed to clarify this issue. Furthermore, analyzing the potential correlations of specific disorders with an approximate assessment of the severity of misophonia, or hyperacusis, or tinnitus (as a control) will highlight which psychological and psychiatric disorders may play a role in misophonia. This approach has been effective in the field of tinnitus before the development of specific, tinnitus-oriented questionnaires.

Results obtained from the above-outlined studies should develop standardized protocols to diagnose misophonia and hyperacusis, with the creation of definitions-based questionnaires for misophonia and hyperacusis. Then, it should be possible to clearly differentiate the presence and severity of misophonia from other coexisting disorders. Having tools to assess specifically the presence and severity of misophonia would allow for the evaluation of the various therapeutical approaches by clinical trials.

Finally, it should be possible to embark on an investigation of physiological mechanisms, which are the basis of misophonia and hyperacusis. Knowledge gained in these investigations would allow for the proposal of new mechanism-based treatments for misophonia.

## Author contributions

PJ and MJ defined the project objectives. PJ devised the theoretical framework of the model and wrote the manuscript. MJ provided feedback on the manuscript. Both authors contributed to the article and approved the submitted version.
